# Paradoxic Tachycardia of Hypertension and the Oculo-Cardiac Reflex

**Published:** 1914-04

**Authors:** 


Paradoxic Tachycardia of Hypertension and the Oculo-Cardiac Reflex. A Mougeot of Royal-les-Bains, *Prog. Med.*, December 20, 1913. The term paradoxic is used on account of the general rule that hypertension slows the heart. As adrenalin also slows the heart by direct action on the muscle, the condition is probably not due to an excess of this secretion in the blood but to an endeavor on the part of the heart to maintain circulatory equilibrium at the point when competence is threatened and would fail if the number of contractions were not increased. The oculo-cardiac reflex consists in a slowing of the pulse when pressure is made on the eyes. The fact that it has been normally present in all but two of the cases of paradoxic tachycardia of hypertension, is evidence that neither the sympathetic nor the pneumogastric is at fault but that the essential trouble is weakness of the left ventricle. In the two exceptions, in which this reflex failed, tabes was present.

